# Determination of the effect of functional single-nucleotide polymorphisms associated with glycerolipid synthesis on intramuscular fat deposition in Korean cattle steer

**DOI:** 10.5194/aab-64-27-2021

**Published:** 2021-01-19

**Authors:** Hyeongrok Kim, Dong-Yep Oh, Yoonseok Lee

**Affiliations:** 1 Department of Biotechnology, College of Agriculture and Life Science, Hankyong National University, Gyeonggi 17579, Republic of Korea; 2 Center for Genetic Information, College of Agriculture and Life Science, Hankyong National University, Gyeonggi 17579, Republic of Korea; 3 Hanwoo's Laboratory, Livestock Research Institute, Gyeongsangbuk-Do, Yeongju, Gyeongbuk 36052, Republic of Korea

## Abstract

Intramuscular fat deposition in the longissimus dorsi muscle (LM) of Korean cattle steer is regulated by several genes related to lipid metabolism. One of these genes encodes the enzyme bovine glycerol-3-phosphate acyltransferase, mitochondrial (*GPAM*), which is located on the mitochondrial outer membrane and catalyzes the initial and committed step of glycerolipid synthesis in lipid metabolism of cattle. Previous studies have shown that the 3${}^{\prime }$-untranslated region (UTR) of the *GPAM* is quite extended and contains a polyadenylation signal site, erythroid 15-lipoxygenase differentiation control elements (15-LOX-DICEs), and cytoplasmic polyadenylation elements (CPEs) that affect the regulation of triacylglycerol synthesis. Therefore, the aim of this study was to identify single-nucleotide polymorphisms (SNPs) related to the regulation of glycerolipid synthesis in the 3${}^{\prime }$-UTR of *GPAM* and to verify the function of SNPs affecting the deposition of intramuscular fat in Korean cattle steer. In the present study, 11 SNPs were discovered in the 3${}^{\prime }$-UTR of *GPAM*. Among these SNPs, g.54853A>G, g.55441A>G, and g.55930C>T were significantly associated with marbling score in a Korean cattle steer population and were strongly correlated with each other within the *GPAM* gene. Furthermore, based on the results predicted by the RNAhybrid program, four putative microRNAs (miRNAs) were identified, and the above SNPs were found to present in the seed region of these miRNAs. These miRNAs have a differential binding affinity for each allele of SNPs g.54853A>G, g.55441A>G, and g.55930C>T. The *in vivo* evidence of intramuscular fat deposition in the LM tissue showed that these SNPs affected the regulation of intramuscular fat deposition in Korean cattle steer. Thus, the g.54853A>G, g.55441A>G, and g.55930C>T could be considered as causal mutations regulating intramuscular fat deposition in Korean cattle steer.

19 January 2021

## 1 Introduction

Intramuscular fat deposition in the longissimus dorsi muscle (LM) of Korean cattle is regulated by several genes related to lipid metabolic processes, such as adipogenesis, lipogenesis, glycerolipid synthesis, and lipolysis. Several previous studies have reported that by increasing the deposition of intramuscular fat in Korean cattle steer, the expression levels of mRNAs related to adipogenesis, glycerolipid synthesis, and lipogenesis were upregulated, whereas those of mRNAs related to lipolysis were downregulated (Jeong et al., 2012; Kim et al., 2008). Among these metabolic processes, the mRNA abundance of the glycerol-3-phosphate acyltransferase 1 (*GPAT1*), which catalyzes the initial and committed step in glycerolipid biosynthesis, showed the greatest correlation with intramuscular fat content (Jeong et al., 2012).

Bovine glycerol-3-phosphate acyltransferase, mitochondrial (*GPAM*), also known as *GPAT1*, is a member of the *GPAT* gene family and is an enzyme that synthesizes lysophosphatidic acid (LPA) by transferring acyl groups to glycerol-3-phosphate (Yu et al., 2017). Thus, this enzyme catalyzes the initial and committed step in glycerolipid biosynthesis and plays a key role in regulating the level of cellular triacylglycerol in cattle (Yu et al., 2017).

The *GPAM* gene is located on bovine chromosome 26q22 and is composed of 21 exons and 20 introns. It is 3689 bp in length and has a much extended 3${}^{\prime }$-untranslated region (3${}^{\prime }$-UTR), which is longer than that of the other bovine genes. Furthermore, the 3${}^{\prime }$-UTR of *GPAM* contains a polyadenylation signal site, erythroid 15-lipoxygenase differentiation control elements (15-LOX-DICEs), and cytoplasmic polyadenylation elements (CPEs) that affect the regulation of triacylglycerol synthesis. Roy et al. (2006) reported that an extended 3${}^{\prime }$-UTR is significant for *GPAM* gene regulation, and a cis-element might be implicated in mRNA stabilization and translational control. The transmembrane domain of the *GPAM* protein is bound to the outer membrane of mitochondria and its N- and C-termini are located in the cytoplasm (Roy et al., 2006).

Recently, Yu et al. (2017) reported that the knockdown of *GPAM* expression significantly reduced the synthesis of triglycerides in bovine embryonic fibroblast (BEF) cells, and the genetic variation of *GPAM* was significantly associated with the fatty acid composition of intramuscular fat in cattle. Furthermore, Roy et al. (2006) reported that the cis-regulatory elements (15-LOX-DICEs and CPEs), which regulate the genes related to lipid degradation and polyadenylation signal, respectively, are located in the 3${}^{\prime }$-UTR of the *GPAM* gene.

Therefore, the aim of this study was to identify SNPs related to the regulation of glycerolipid synthesis in the 3${}^{\prime }$-UTR of *GPAM* and to verify the function of SNPs affecting intramuscular fat deposition in Korean cattle steer.

## 2 Materials and methods

### 2.1 Animals, DNA extraction, SNP discovery

Animal welfare issue was followed according to approved guidelines of the Animal Care and Use Committee of Hankyong National University. LM tissue samples were collected from Korean cattle (n=192) raised in Pyeongchang (Gangwon, Republic of Korea). The marbling grade of this beef was classified according to the carcass grading standard of the Korea Institute of Animal Products Quality Evaluation (KAPE, 2017). The KAPE provided grades for beef marbling standard (BMS) score. All steers were maintained under constant environmental conditions, with two types of commercial feeds in six feedlots. Genomic DNA was extracted from the LM tissue using a LaboPass™ tissue mini kit (Cosmo Genetech, Seoul, Republic of Korea). In order to discover SNPs, the 3${}^{\prime }$-UTR sequence of *GPAM* was obtained from the National Center for Biotechnology Information (NCBI) GenBank database (accession no. NC_037353.1). The primers were designed using NCBI Primer-BLAST based on the selected polymorphism sites, and the primer sequences are shown in Table S1 in the Supplement. The sequencing was performed according to a previous study (Lee et al., 2010), and SNPs were discovered using the “SNP Hunting” option of the Sequencer v5.2.4 program (Gene Codes Corp., Ann Arbor, MI, USA). In order to map the functional SNPs on DNA, mRNA (NM_001012282.1), and protein (NP_01011282.1), these sequences were aligned using the NCBI graphical sequence viewer. The correlation coefficients between SNP pairs were calculated by Haploview 4.1 (Broad Institute, Cambridge, MA, USA) with genotypes of the *GPAM* gene in Korean cattle.

**Table 1:** Effect of SNPs in the 3${}^{\prime }$-UTR of the *GPAM* gene on marbling scores in Korean cattle steer (n=190).

SNP	Genotype (n)			p value
	LSM${}^{1}$ ± SE${}^{2}$			
g.53373C>T	CC (90)	TC (79)	TT (20)	0.424
(rs208584618)	5.882 ± 0.216	6.215 ± 0.223	6.025 ± 0.435	
g.54193C>T	CC (104)	TC (74)	TT (11)	0.486
(rs207798182)	6.002 ± 0.209	6.147 ± 0.234	5.808 ± 0.551	
g.54316A>C	AA (72)	AC (87)	CC (30)	0.015
(rs210457037)	5.637 ± 0.228${}^{a}$	6.328 ± 0.209${}^{b}$	5.918 ± 0.332${}^{b}$	
g.54559T>C	TT (6)	TC (44)	CC (140)	0.765
(rs208546590)	5.383 ± 0.690	6.442 ± 0.310	6.074 ± 0.193	
g.54597A>T	AA (143)	AT (41)	TT (5)	0.825
(rs210916913)	6.042 ± 0.186	6.432 ± 0.312	5.477 ± 0.749	
g.54853A>G	AA (51)${}^{a}$	AG (77)	GG (61)	0.013
(rs134324818)	5.640 ± 0.289	6.418 ± 0.238${}^{b}$	5.988 ± 0.282${}^{b}$	
g.55441A>G	AA (50)	AG (78)	GG (61)	0.013
(rs207643468)	5.638 ± 0.291${}^{a}$	6.384 ± 0.238${}^{b}$	5.989 ± 0.283${}^{b}$	
g.55517G>A	GG (115)	GA (64)	AA (9)	0.362
(rs380698026)	6.034 ± 0.198	6.102 ± 0.237	5.968 ± 0.592	
g.55930C>T	CC (50)	CT (79)	TT (60)	0.010
(rs133256691)	5.644 ± 0.296${}^{a}$	6.400 ± 0.235${}^{b}$	5.987 ± 0.286${}^{b}$	
g.56493G>A	GG (104)	GA (74)	AA (11)	0.486
(rs210017870)	6.002 ± 0.209	6.147 ± 0.234	5.808 ± 0.551	
g.56806A>G	AA (36)	AG (73)	GG (79)	0.212
(rs137535375)	5.804 ± 0.345	6.345 ± 0.247	5.904 ± 0.244	

${}^{a,b}$ Means with the same superscript in the same row for each quality are not significantly different (p<0.05). ${}^{1}$ LSM: least square mean. ${}^{2}$ SE: standard error.

### 2.2 SNP genotyping and statistical analysis

SNPs were genotyped commercially using the Fluidigm^®^ SNP™-type assay platform according to a previous study (Oh et al., 2018). In order to evaluate the association SNPs and carcass traits, these data were analyzed using a generalized linear model (GLM) in SPSS v22 (IBM, Chicago, IL, USA) with the following equation:

1 $$Y_{ijkl}=\mu +{\mathrm{Farm}}_{i}+{\mathrm{Sire}}_{j}+{\mathrm{SNP}}_{k}+\beta_{\mathrm{age}}+e_{ijkl},$$

where $Y_{ijkl}$ is the observed marbling score of Korean cattle; μ is the overall mean; SNP${}_{k}$ is the fixed effect of SNP genotype or haplotype; Farm${}_{i}$ is the fixed effect of the feed type in farm; Sire${}_{j}$ is the random effect of the sire; $\beta_{\mathrm{age}}$ is the covariation of age; and $e_{ijkl}$ is random error. The correlation coefficient between SNP pairs in the 3${}^{\prime }$-UTR of *GPAM* was analyzed using the Haploview program (Broad Institute, USA). The relationship between SNPs and beef quality grades (1${}^{++}$ and 2) was analyzed using Fisher's exact test in SPSS v22 (IBM, USA).

### 2.3 Bioinformatics analysis of target microRNAs

In order to identify the microRNAs (miRNAs) that bind to candidate functional SNPs in the 3${}^{\prime }$-UTR of *GPAM*, the TargetScan (http://www.targetscan.org, last access: 15 November 2019) and miRNA_Target (Kumar et al., 2012) software programs were used. Subsequently, their sequences were obtained from miRBase (http://www.mirbase.org, last access: 15 November 2019). The RNAhybrid program (http://bibiserv.techfak.uni-bielefeld.de/rnahybrid, last access: 15 November 2019) was employed to calculate the minimum free energy (MFE) of binding between miRNAs and their alleles, and the threshold was selected according to a previous study (Knox et al., 2018; Rehmsmeier et al., 2004).

## 3 Results

### 3.1 Identification of SNPs and their functional characterization

**Figure 1:**
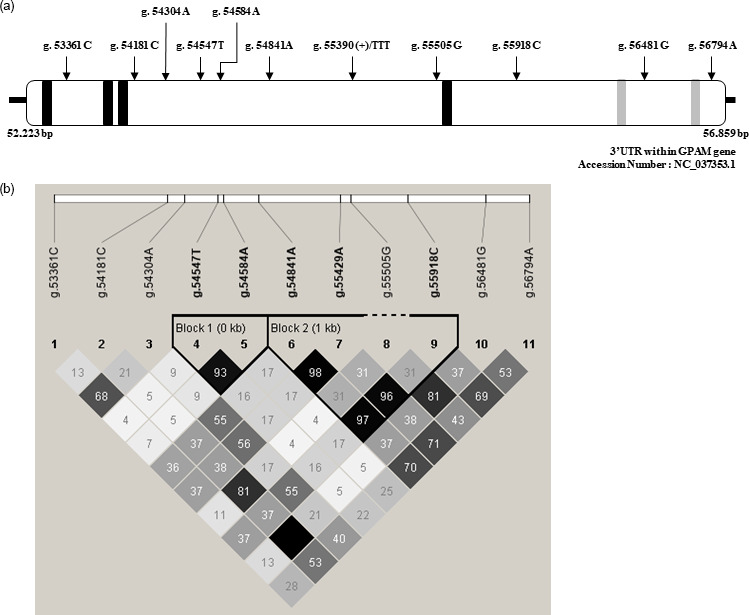
Positions of single-nucleotide polymorphisms (SNPs) in the 3${}^{\prime }$-UTR of the *GPAM* gene and their pairwise correlation. **(a)** SNP position in the 3${}^{\prime }$-UTR of the GPAM gene. A total of 11 SNPs, 15-LOX-DICE and CPE, which are cis-regulatory elements, were mapped in the 3${}^{\prime }$-UTR of Korean cattle steer *GPAM*. 15-LOX-DICE and CPE are represented by black and grey boxes, respectively. **(b)** Pairwise SNP correlation. The color code on the Haploview plot follows the $r^{2}$ color scheme: white ($r^{2}=0$); shades of grey ($0<r^{2}<1$); black ($r^{2}=1$). The numbers in the cells are the $r^{2}$ values. However, the $r^{2}$ value of 1.0 is not shown (empty cell).

In the present study, we discovered 11 SNPs within the 3${}^{\prime }$-UTR of *GPAM* in a Korean cattle steer population using the direct sequencing method. The positions of SNPs within the 3${}^{\prime }$-UTR of *GPAM* and their pairwise correlation coefficients are shown in Fig. 1. As shown in Fig. 1a, although the detected 11 SNPs were present in the 3${}^{\prime }$-UTR of *GPAM*, they were not specifically present in the sequences of four 15-LOX-DICEs and two CPEs present in the 3${}^{\prime }$-UTR. For determining linkage disequilibrium (LD) among 11 polymorphic SNPs in the 3${}^{\prime }$-UTR of *GPAM*, we calculated the correlation coefficient between SNP pairs using the Haploview software. The correlation coefficient between SNP pairs is shown in Fig. 1b. As shown in Fig. 1b, two LD blocks were detected in the 3${}^{\prime }$-UTR of *GPAM*. The LD block structure included two SNPs, whereas the other structure included four SNPs. Three SNPs, g.54853A>G, g.55441A>G, and g.55930C>T, were strongly correlated with each other, except for the g.55517G>A SNP.

Previous studies have reported that the 3${}^{\prime }$-UTR of the *GPAM* gene is longer than the open reading frame (ORF) region and includes a cis-element that plays a key role in mRNA stabilization and translational control (Roy et al., 2006; Yu et al., 2017). Furthermore, Yu et al. (2017) showed that the SNPs in the 3${}^{\prime }$-UTR of *GPAM* were significantly associated with fatty acid composition of intramuscular fat and marbling score in a beef cattle population.

### 3.2 Association of functional SNPs with marbling score

In order to evaluate the function of 11 SNPs in the regulation of glycerolipid synthesis, we analyzed the association between these SNPs and marbling score. The effects of these SNPs and their combinations on the marbling score of Korean cattle are shown in Tables 1 and 2.

**Figure 2:**
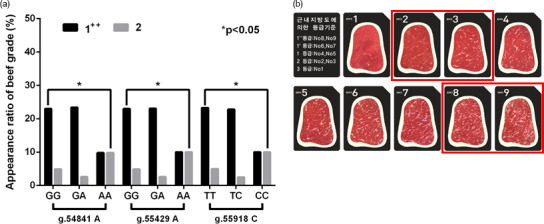
*In vivo* evidence of the genotype effect of functional SNPs in the 3${}^{\prime }$-UTR of *GPAM* on the beef quality in Korean cattle steer. **(a)** Association of candidate functional SNPs with Korean cattle steer beef quality grade. **(b)** Grade system of beef quality.

As shown in Table 1, four SNPs (g.54316A>C, g.54853A>G, g.55441A>G, and g.55930C>T) were significantly associated with the marbling score of the Korean cattle steer population. The marbling scores in group with the heterozygote genotypes of SNPs g.54316A>C, g.54853A>G, g.55441A>G, and g.55930C>T were significantly higher than those with the homozygote genotypes of these SNPs. Furthermore, as shown in Table 2, the combination types of SNPs g.54853A>G, g.55441A>G, and g.55930C>T were significantly associated with the marbling score of the Korean cattle steer population. Especially, the group with the combination types GA, GA, and TC had the highest average marbling score in the studied Korean cattle steer population.

**Table 2:** Effect of combination SNPs on marbling scores in Korean cattle steer (n=189).

Block	Combination	No. of	Marbling	p value
	type	animals	LSM ± SE	
Block 1	CC-AA	140	6.129 ± 0.176	0.918
	CT-AT	41	6.355 ± 0.302	
	CT-AA	3	7.000 ± 0.960	
	TT-TT	5	5.400 ± 0.743	
Block 2	GG-GG-TT	60	6.019 ± 0.269	0.044
	GG-GG-TC	1	6.000 ± 1.749	
	GA-GA-TC	77	6.433 ± 0.234	
	AA-GA-CC	1	5.000 ± 1.749	
	AA-AA-TC	1	6.000 ± 1.749	
	AA-AA-CC	49	5.628 ± 0.295	

### 3.3 miRNA prediction and its target alteration by SNP alleles

In the present study, we predicted miRNAs that bind to candidate functional SNPs using a bioinformatics tool, in order to determine whether these SNPs had an effect on the regulation of glycerolipid synthesis. We identified four miRNAs that bind to candidate functional SNPs present in the seed region of miRNA using the online software programs TargetScan and miRNA_Target (Table 3). To increase the credibility of our results, we used the RNAhybrid software to quantitatively determine the binding affinity between miRNAs and SNPs located in the seed region.

**Table 3:** Information of miRNAs that bind on candidate functional SNPs in the 3${}^{\prime }$-UTR of the *GPAM* gene.

SNP	Allele type		MAF1${}^{1}$	miRNA	MFE2${}^{2}$ (kcal mol${}^{-1}$)		Seed region type
	Major	Minor			Major	Minor	
g.54853A>G	G	A	0.474	bta-miR-2418		-21.3	7mer-m8
g.55441A>G	G	A	0.471	bta-miR-375		-18.9	7mer-m8
				bta-miR-2479		-11.9	7mer-A1
g.55930C>T	T	C	0.474	bta-miR-2468		-19.8	7mer-m8

${}^{1}$ MAF: minor allele frequency. ${}^{2}$ MFE: minimum free energy.

As shown in Table 3, three SNPs (g.54853A>G, g.55441A>G, and g.55930C>T) were predicted to bind to four miRNAs (bta-miR-2418, bta-miR-375, bta-miR-2479, and bta-miR-2468) according to the seed region type. The seed region or seed sequence, which is two to seven nucleotides long at the 5${}^{\prime }$ end of the miRNA sequence, is essential for the binding of the partially complementary miRNA to the mRNA. The seed region is classified into atypical sites, canonical sites and marginal depending on the number of nucleotides matching between the seed sequence and the mRNA (Bartel, 2009). Another important factor on which miRNA binding depends is whether is adenine or guanine is the first nucleotide at the 5${}^{\prime }$ end of miRNA (Bartel, 2009). In addition, the binding efficiency is in the order of (a) 8mer, (b) 7mer-m8, (c) 7mer-A1, and (d) 6mer. As shown in Table 3, the seed region types of the miRNAs bta-miR-2418, bta-miR-375, and bta-miR-2468 were all 7mer-m8, except for bta-miR-2479.

Especially, to increase the binding efficiency, we analyzed the G-U wobble base pair parameter using the RNAhybrid software. The binding energies of the miRNAs that bind to the sites with major alleles of the three SNPs were all zero. As a result of the minor alleles shown in Table 3, the binding energy of the miRNA-mRNA seed region with an A allele of SNP g.54853A>G was the lowest at -21.3 kcal mol${}^{-1}$, whereas the binding energy between the A allele of SNP g.55441A>G and miRNA bta-miR-2479 was the highest at -11.9 kcal mol${}^{-1}$.

### 3.4 *In vivo* evidence of the genotype effect of functional SNPs on marbling score

To validate the effect of these functional SNPs on intramuscular fat deposition *in vivo*, we determined the relationship between three functional SNPs and Korean cattle beef grades (Fig. 2). As shown in Fig. 2, three candidate functional SNPs (g.54853A>G, g.55441A>G, and g.55930C>T) caused a significant difference between beef grades “1${}^{++}$” and “2”. As shown in Table 3, the binding affinities of the minor alleles of candidate SNPs were higher than those of their major alleles. Thus, these results suggested that the miRNA-minor alleles of these SNPs act as inhibitors in the regulation of glycerolipid synthesis.

As a result of the relationship between these SNPs and beef grades, as shown in Fig. 2, we identified that the “2” beef grade in the group with the minor alleles of these SNPs was significantly lower than that in the group with major alleles (p<0.05).

## 4 Discussion

In the present study, we evaluated whether the 3${}^{\prime }$-UTR of *GPAM* was influenced by the regulation of glycerolipid synthesis. The 3${}^{\prime }$-UTR of *GPAM* is 3689 bp in length and is longer than those of the other genes (Chen et al., 2012). The 3${}^{\prime }$-UTR has an important influence on the regulation of gene expression because a potential miRNA response element (MRE) is present in this region (Arnold et al., 2012).

Recently, Yu et al. (2017) reported that an SNP in the 3${}^{\prime }$-UTR of *GPAM* was significantly associated with fatty acid composition of intramuscular fat and marbling score in a beef cattle population. In the present results, as shown in Tables 1 and 2, we demonstrated that SNPs g.54316A>C, g.54853A>G, g.55441A>G, and g.55930C>T, which are located in the 3${}^{\prime }$-UTR, were associated with marbling score in the studied Korean cattle steer population. These results coincide with those reported by Yu et al. (2017). Furthermore, as *in vivo* evidence of the genotype effect of functional SNPs on marbling score, the group with the major alleles of these SNPs had significantly lower binding affinities between the seed region allele and miRNA than those with the minor alleles of these SNP. Thus, these results suggested that these SNPs had an important effect on the regulation of glycerolipid synthesis in the studied Korean cattle steer population.

We found that the 3${}^{\prime }$-UTR of the *GPAM* gene contained four 15-LOX-DICEs and two CPEs, which regulate mRNA translation and structure stabilization. However, no SNPs were detected in the region containing four 15-LOX-DICEs and two CPEs. In cattle, miRNA targeting has predominantly been associated with the 3${}^{\prime }$-UTR region of the transcripts derived from ORFs, typically leading to down-regulation through triggering RNA degradation, RNA instability, and/or reduction (He and Hannon, 2004; Li et al., 2011). Thus, as shown in Table 3, four miRNAs were predicted to bind to the seed region including SNPs g.54853A>G, g.55441A>G, and g.55930C>T, using the software programs TargetScan and RNAhybrid. According to the minimum free energy of the binding affinity, each allele of these SNPs had a different binding affinity. Furthermore, this difference in binding affinities led to a significant difference between beef grades “1${}^{++}$” and “2”. Thus, our results suggested that the three SNPs, which are present in the 3${}^{\prime }$-UTR, could be genetic variations influencing the regulation of *GPAM* gene expression.

## 5 Conclusions

Glycerol-3-phosphate acyltransferase, mitochondrial, the enzyme located on the mitochondrial outer membrane, catalyzes the initial and committed step in glycerolipid synthesis in the lipid metabolism of cattle. In the present study, we determined whether the SNPs located in the 3${}^{\prime }$-UTR of *GPAM* affect the regulation of gene expression. Of the 11 SNPs detected in the 3${}^{\prime }$-UTR of *GPAM*, three SNPs, g.54316A>C, g.54853A>G, g.55441A>G, and g.55930C>T, were significantly associated with marbling score in Korean cattle steer population and showed strong pairwise correlation. Furthermore, we identified four putative miRNAs and found that these SNPs were present in the seed region of these miRNAs. These miRNAs showed a differential binding affinity for each allele of SNPs g.54316A>C, g.54853A>G, g.55441A>G, and g.55930C>T, leading to a significant difference between beef quality grades “1${}^{++}$” and “2”. Thus, our results suggested that three SNPs in the 3${}^{\prime }$-UTR could be genetic variations affecting the regulation of *GPAM* gene expression.

## Supplement

10.5194/aab-64-27-2021-supplement The supplement related to this article is available online at: https://doi.org/10.5194/aab-64-27-2021-supplement.

## Data Availability

The original data of the paper are available from the corresponding author upon request.
