# Supplementary material for: Determination of the effect of functional single-nucleotide polymorphisms associated with glycerolipid synthesis on intramuscular fat deposition in Korean cattle steer
Source: Arch Anim Breed. 2021 Jan 19;64(1):27–33. doi: 10.5194/aab-64-27-2021 (PMC8130544; doi:10.5194/aab-64-27-2021)
Supplement: The supplement related to this article is available online at: https://doi.org/10.5194/aab-64-27-2021-supplement. [file aab-64-27-supplement.pdf]

Supplement of Arch. Anim. Breed., 64, 27–33, 2021
https://doi.org/10.5194/aab-64-27-2021-supplement
© Author(s) 2021. This work is distributed under
the Creative Commons Attribution 4.0 License.

Open Access
Archives Animal Breeding

Supplement of

Determination of the effect of functional single-nucleotide polymorphisms associated with glycerolipid synthesis on intramuscular fat deposition in Korean cattle steer

Hyeongrok Kim et al.

Correspondence to: Yoonseok Lee (yoonseok95@hknu.ac.kr)

The copyright of individual parts of the supplement might differ from the CC BY 4.0 License.

Supplementary Table S1. Primer sequences for direct sequencing in 3′-UTR of *GPAM*.

Primer name		Primer sequence	Product size (bp)	Annealing temperature (℃)
Primer 1	Forward	CCTAAATGAAGGTGTCCATGTG	705	58
	Reverse	AAGGCACTTGTCTTCCAGGAG		
Primer 2	Forward	TGTGGTGCTGTAGGTAACTTCG	701	
	Reverse	TTCCTGGTACAAGCTGACTGGT		
Primer 3	Forward	GGTGTCACTGTGCTGTCTGTTG	690	
	Reverse	TGAACAATTACGATCCCTGCTC		
Primer 4	Forward	CGGACTGGCATTTGTAGATGTT	603	
	Reverse	TAACACAGTGAAGATCCCACCC		
Primer 5	Forward	CCGTTGTGATTTTCTTCCACTC	690	
	Reverse	TGGTATCTGGAAGCCTTACTGG		
Primer 6	Forward	CCAAAGTTCTGCTCATCCAGAC	673	
	Reverse	CCTGAGCCCTTACTGTGCTAAA		
Primer 7	Forward	TCCGTGTGTACGAAGGCTCTAT	674	
	Reverse	CAGATGCCAAGTCTCAAGTTCC		
Primer 8	Forward	AGACCTGGCAGAAGACATTGC	589	
	Reverse	GCACTGCAGGTTCACAAATGTA		
